# Dr. Hamett's Official Medical Reports on Cholera

**Published:** 1833-01-01

**Authors:** 


**THE SUBSTANCE OF THE OFFICIAL MEDICAL REPORTS UPON THE EPIDEMIC CALLED CHOLERA, WHICH PREVAILED AMONG THE POOR AT DANTZICK, BETWEEN THE END OF MAY AND THE FIRST PART OF SEPTEMBER, 1831, AS TRANSMITTED TO THEIR LORDSHIPS, &c. &c. By *John Hamett*, M.D. Octavo, pp. 190. (With a Map.) Highley, 1832.**

OUR readers are aware that in No. 31 of this Journal, for January, 1831, we inserted a long paper, containing extracts from this Official Report of Dr. Hamett, not then published, and which we accidentally got possession of. The whole Report is now before the public, and is highly deserving of their attention. For the above reason it will not be necessary for us to do much more than announce the publication of the work, our former paper being an analysis of its contents. We deem it proper, however, to observe that the intelligent and zealous author was commissioned by Government, in June 1831, to proceed to Dantzick, in order to investigate and report upon the epidemic raging in that city. He did so; and discharged his duty with equal assiduity and talent. But, alas! Dr. Hamett is not a "man of the world!" Had one of the contagion-hunters of London gone to Dantzick, he would have returned with a budget of stories, dressed up for the "Powers that be," or rather the powers that were, filled with all manner of tales and hearsays tending to keep up quarantine restrictions, and elevate the crests of the *Doctrinaires!* But Dr. Hamett was an honest man—that is to say, a fool for his own interest, (no man should think of the public when *himself* is concerned,) and reported the truth to the Privy Council. The Lords of the Privy Council could be small judges of the matter, and as his Reports were necessarily referred to other tribunals, it was easy to foretel their fate.

The author was marked as a black sheep—

Hic *niger* est—hunc tu Romane caveto was written upon the Official Report—and one of the most important documents was—no more! It happened, however, that Dr. Hamett took the precaution of having them copied and authenticated at Dantzick, otherwise the missing paper would never have seen the light.

"On arriving in London, I learned that a Committee of the College of Physicians had received instructions to draw up, in a concise form, the facts in my Reports connected with the Epidemic at Dantzick, together with the description and treatment of the disease. This task the College transferred to myself, and my Reports were directed to be given up to me, accordingly. I received all back, except *Medical Report A*, containing a mass of circumstantial evidence, in support of my conclusions, that the disease had not been imported into Dantzick, and that it did not prove contagious in that city. This Report, it will be proper to state here, comprised certain authentic isolated cases of Cholera, and the four first acknowledged cases of the Epidemic, which had all occurred previously to the first arrival of vessels from Russian ports, together with authentic communications from the physicians in charge of the different Cholera Hospitals, and from Dr. Baum, Physician-General of the Town Hospital. This important document I was unable to recover, although I made proper inquiry for it.

It appeared that the Board of Health, instituted in the beginning of the Summer of 1831, had duly received the medical report in question; but that, at the time of my arrival in London, it was not in possession of the Committee of the College of Physicians,—subsequently instituted, and at the same time, I believe, with the Central Board of Health,—as will appear from the following extract of a letter, dated Nov. 30th, 1831, from the Secretary of the Committee of the College to me:—

'I have to regret extremely that I did not send you Medical Report A before you quitted London. One of the Members of the Board, who did not hear it read publicly, took it home, and never returned it,—and I have been unable to recover it. I must again express my great regret in having exposed you to additional trouble.'

The Committee of the College would, I need not say, most certainly have delivered up that Report with the rest, had it been in their possession.

In a question of such vital importance as that of the contagion of Cholera, it is not a little singular that such a document should be thus missing. Had any accident happened to me whereby my papers had been lost on my passage home, or had I left Dantzick without getting the rough drafts of my Reports authenticated, which I might have done but for the friendly and judicious advice of Mr. Gibsone, the British Consul there, my Reports, with only the remaining facts furnished in support of my conclusions, though highly important in themselves, must appear deficient.—The isolated cases, and the four first cases of the Epidemic, contained in that Report, I have inserted in the history of the disease; and the communications from the physicians before-mentioned, in the article of *the Question of the Cantagion of Cholera at Dantzick, considered solely from Facts.*

Finding, on my arrival, that the principle of contagion had been adopted by Government—and as my details upon the subject, with the inferences drawn from them, were decidedly in opposition to that principle, I had some reason to apprehend that such extracts as I had to make from my Official Reports, would not be published,—still, as containing so many facts of high importance to the public, I imagined that they would not be overlooked.

Having, without delay, made out Extracts from my Official Reports, 'they were approved of, [as I was assured by authority,] without one dissentient voice, by the Committee of the College of Physicians,'—and recommended to the Privy Council in the following terms:—

'The Committee of Physicians, to whom the Reports of Dr. Hamett upon the *Cholera Spasmodica* at Dantzick were submitted by His Majesty's Privy Council, have approved of the accompanying Extracts, and recommend the printing of them for the public information, as a very valuable addition to the present knowledge of the disease, procured by great diligence and painful and unremitted observation.'

From this official recommendation, it evidently appears that the Committe of the College behaved in a liberal,—as, indeed, they previously had in a courteous manner to me. Pursuant to this recommendation their Lordships ordered my Extracts to be printed,* and a proof copy was, in due time, sent to the Superintendent-General of Quarantine. But, notwithstanding the said recommendation and consequent order, they were not published,—and yet, however paradoxical it may seem, they were not suppressed by Authority.

---

* "An attempt was now made by the Superintendent-General of Quarantine to induce me not to print the authorized Extracts above-mentioned."

After the first rough copy had been printed, it was in vain that I waited in expectation of hearing of an order being given for their publication. On one occasion, indeed, it was indirectly intimated to me by the Superintendent-General of Quarantine, that the Privy Council did not sanction their publication,—but this, not only from what I have just stated, but according to what I have since learned from authority, could not have been the case. The reason, ultimately given for delay in the publication by the publisher of the Official Reports upon Cholera to Government, was, that it was intended to bind up and circulate them with the second edition of the St. Petersburg Reports, not completed. This was rather an unfair arrangement, inasmuch as the facts and results of the two Reports were diametrically opposite, and inasmuch as the contents of the latter were apparently greater; while the comparative labour and observation bestowed upon each could not be readily perceived, except by such persons as are acquainted with the various consequential and inductive steps necessary to be pursued in the investigation of epidemics."—*Introd. p.* xi.

Such are the tactics of the *ultra*-contagionists in this country—and such have been the tactics throughout every ramification of that faction, from the metropolis down to the dirtiest village of Scotland and Ireland. How we have resisted the demoralizing doctrines of quarantines and contagion is well known to our readers. The above extract contains a sample, and a very *favourable* one, of their manœuvres in a bad cause. As such we have recorded it, for the edification of the present and future generations.

It is some—but a small consolation, to find that our worthy, not worldly, author has had the satisfaction of receiving the following brief and official acknowledgment of his zeal and diligence.

"Council Office, Whitehall,
25th August, 1832.

SIR,

I am directed by the Lords of His Majesty's Most Honourable Privy Council to inform you, that they will accept with pleasure the dedication, which you are desirous of making to them of your Medical Reports on Cholera; and their Lordships take this opportunity of renewing to you the assurance of their satisfaction at the diligence and zeal, which you evinced in the performance of the very arduous duty imposed upon you, in your Mission to Dantzick, in June, 1831.

I am, Sir,

Your obedient Servant,

C. C. GREVILLE."

To Dr. Hamett.

To attempt *an analysis* of the present volume, would be to repeat the whole of what we published in a former Number, with additions. We can only, therefore, recommend our readers to patronize the author, who has gone to a great personal expense, by procuring the work; or, at all events, by proposing it in the various book-societies throughout the kingdom.

There is a long chapter (XII.) appended to this edition, entitled "the Question of the Contagion of Epidemic Cholera more fully considered," which we would strenuously recommend to the perusal of contagionists and anti-contagionists. It is a very able document—and may serve to confirm the creed of the latter party, and mitigate the confidence of the former.

